# Mapping of the chicken *cleft primary palate* mutation on chromosome 11 and sequencing of the 4.9 Mb linked region

**DOI:** 10.1111/age.12927

**Published:** 2020-03-12

**Authors:** I. A. Youngworth, M. E. Delany

**Affiliations:** ^1^ Department of Genetics Stanford University Stanford CA 94305 USA; ^2^ Department of Animal Science University of California Davis Davis CA 95616 USA

**Keywords:** capture array, cleft palate, development, ESRP2, facial truncation, SNP genotyping

## Abstract

An embryonic lethal mutation in chicken named *cleft primary palate *(*cpp*) is inherited in an autosomal recessive mode and results in a severely truncated upper beak. In this study, genotyping and sequencing techniques were employed to advance our genetic and genomic knowledge of the mutation’s chromosomal location, candidate region and possible causative element using a congenic inbred line. Herein, the candidate region for the *cpp* developmental mutation was established as a ca. 5.1 Mb region of chicken chromosome 11 (GGA 11) through the use of a 600K Affymetrix SNP array. The SNPs identified from this array linked to *cpp* were used to genotype individuals from the congenic inbred line over several generations and thereby fine‐map the causative region resulting in an approximately 200 kb size reduction. This candidate region (4.9 Mb) was sequenced via capture array in a cohort of 24 individuals, including carriers, mutants and their wild type (wt) siblings. Interestingly, the GGA 11 region for *cpp* encompasses the predicted centromere location and is thus unlikely to be highly disrupted by further recombination. Here we report on the variation unique to the *cpp* mutation, i.e. single‐nucleotide variants and insertions or deletions. Although the candidate region contains several genes of interest with regard to the *cpp* phenotype, only one *cpp*‐linked variant was predicted to have a significant physiological effect by causing a frameshift mutation in *ESRP2*, which has a role in tissue‐specific splicing during development.

## Introduction

The intricacies of facial development have long held interest for researchers as a high incidence of spontaneous congenital facial malformations in humans is observed worldwide. Craniofacial anomalies, such as clefting of the lip and/or palate, may be caused by chemical or environmental factors during embryonic development and can be due to genetic factors as well (Schutte & Murray 1999; Cobourne 2004; Schock *et al.* 2016). The chicken is a valuable animal model for the study of vertebrate development for reasons such as its accessibility even at extremely early stages of embryogenesis, short generation time, size and ability to tolerate inbreeding (Delany 2004). Thus, a number of congenic inbred lines retaining specific developmental mutations have been developed in chicken to advance research of the mutations and their phenotypes (Abplanalp 1992; Pisenti *et al.* 1999). *Cleft primary palate* (*cpp*) is one such mutation in a congenic inbred line, UCD Cleft primary palate.003 (UCD cpp.003).

The *cpp* mutation results in a severely abnormal frontonasal prominence, which gives the appearance of a recessed and upturned upper beak (see figure 4P‐T in Schock *et al.* 2016). This tissue defect appears to start with the abnormal fusion of facial prominences, coincident with and possibly owing to the absence of prefusion filaments in the mutants (Yee & Abbott 1978; Youngworth 2019). The *cpp* mutation is inherited in an autosomal recessive fashion with complete penetrance and is embryonic lethal between 17 and 20 days of incubation (Abbott & MacCabe 1966). The mutation was first described under the name *ectrodactyly *(*ec*) and arose in a line carrying another autosomal recessive mutation named *scaleless *(*sc*)*.* The homozygous *sc* mutation, as its name implies, results in absence of scales, as well as foot pads, spurs and most feather follicles, but otherwise does not cause craniofacial or limb defects (Abbott & MacCabe 1966). The double *sc*/*ec* mutants were observed with these *sc* defects as well as an absent upper palate and abnormal hindlimbs (Abbott & MacCabe 1966). Outcrosses of the *sc*/*ec* carriers to normally feathered birds produced carriers who were test mated and then crossed to produce mutants with the palate abnormality alone to show that the mutations were unlinked (notably, the hindlimb abnormality occurred only in the double *sc/ec* mutants). The *ec* mutation was later backcrossed for nine generations into a highly inbred Single Comb White Leghorn line (UCD 003) and renamed *cleft primary palate* to reflect the key phenotype of the single mutation. The line was then closed to create the separate congenic inbred line UCD cpp.003, estimated to have greater than 99% DNA identity to UCD 003 outside of the introgressed mutation‐encoding region given the number of backcrosses of the *cpp*‐containing line to the highly inbred UCD 003 background line (Abplanalp 1992). Birds of the UCD cpp.003 line are test mated to verify carrier status prior to annual reproduction. The fact that carriers are phenotypically normal with consistent Mendelian ratios of mutants to carriers and non‐carriers shows that the mutation is probably a recessive single‐gene defect (Abbott & MacCabe 1966).

Research reported here applied genetic and genomics methods to map and fine‐map the mutation, as well as identify and narrow the list of potential causative elements. Use of an Affymetrix 600K SNP array (Kranis *et al.* 2013) provided the first new genetic data about the mutation beyond its mode of inheritance and mapped it to chicken chromosome 11 (GGA 11) within a region of ca. 5.1 Mb per the Gallus_gallus‐5.0 genome build (galGal5). Additional analysis at several linked SNPs was subsequently used to genotype carrier individuals and fine‐map the mutation to a smaller region, ca. 4.9 Mb, notably encompassing the predicted location of the centromere. A capture array and next generation sequencing were then employed to sequence this region in a cohort of carrier, mutant and wild type (wt) individuals from the UCD cpp.003 line to create a high‐resolution list of potentially causative variants. Based on predictive functional analyses of all of the variants the only unique, linked variant predicted to have a severe physiological effect was a single‐base deletion creating a frameshift mutation in *ESRP2*. *ESRP2*, or epithelial splicing regulatory protein 2, is an RNA‐binding protein that mediates alternative splicing in development, such that tissue layer‐specific splicing in epithelium and mesenchyme is achieved (Warzecha *et al.* 2009a,b; Dittmar *et al.* 2012).

## Materials and methods

### Sample collection

Samples were derived from the UCD cpp.003 developmental congenic inbred chicken line. As is standard, phenotypically normal heterozygous carriers were bred *inter se* for test mating and reproduction in accordance with an approved animal care protocol (no. 18816). Mutant individuals were obtained from these crosses after 11 days of incubation, or at stage 36 HH of development (Hamburger & Hamilton 1992), as the abnormal craniofacial phenotype of the mutants is unmistakable by this time. DNA was isolated from blood or extraembryonic membrane tissue using the DNeasy Blood and Tissue Kit (Qiagen).

### 600K SNP array

DNA samples from nine *cpp* UCD cpp.003 mutants (*cpp/cpp*) produced by the 2010 carrier generation and two UCD 003 birds were analyzed by utilizing an Affymetrix 600K SNP array (Kranis *et al.* 2013).

### SNP genotyping

SNPs from the 600K array at either end of the linked region were used for further genotyping. Primers were designed to amplify the SNP and flanking DNA by PCR (Table S1). This was first performed in a small test set of birds from the 2014 generation (two +/*cpp* carriers, two *cpp/cpp* mutants and two UCD 003 wt birds) and then in two generations of carrier birds (*n* = 14 in 2015, and *n* = 11 in 2016). When a SNP was observed to be unlinked in a bird, additional SNPs were genotyped to refine the putative recombination breakpoint.

### Capture array and next generation sequencing

Genomic DNA samples from 24 genotyped individuals (13 +/*cpp* carriers, nine *cpp/cpp* mutants and two +/+ wt siblings) were sent to QB3 Vincent J. Coates Genomics Sequencing Laboratory (University of California, Berkeley) for performing library preparation with KAPA library prep kits (Kapa Biosystems). Samples were multiplexed into sets of 12. The capture reaction was performed with the multiplexed samples using a NimbleGen SeqCap EZ kit with custom tiled probes (Roche) to select 4.9 Mb of GGA 11. Sequencing was performed on two MiSeq lanes, using a 75 PE V3 chemistry kit (Illumina), 12 samples per lane.

### Bioinformatics

The initial quality of the raw read data was determined using fastqc (version 0.11.7; Andrews 2010). Trimmomatic (version 0.38; Bolger *et al.* 2014) was used to trim adapters and remove low quality reads. bowtie2 (version 2.3.4.3; Langmead & Salzberg 2012) was used to align to Gallus_gallus‐5.0 (2015 release, NCBI ID: 595851). samtools (version 1.9; Li *et al*. 2009) was used to convert sam alignment files to binary (bam), sort and merge these, and generate pileup files. Bedtools (version 2.27.0; Quinlan & Hall 2010) was used to assess read coverage in the region. Picard (version 2.18.22; Broad Institute 2018) was used to mark duplicates, varscan (version 2.3.9; Koboldt *et al.* 2012) identified SNVs and short indels (1–30 bp range; Koboldt *et al.* 2013) and vcftools (version 0.1.12b; Danecek *et al.* 2011) was used for filtering the vcf files by region. Across the 24 samples, average coverage was 20 reads per base (20×) over the region (with the exception of the predicted centromere). On average, a sample had 450 kb with fewer than eight reads and 50 kb out of this had zero reads. To maximize the coverage and therefore detectable variation, we analyzed the mutants as a pool (*n* = 9) and the carriers as a pool (*n* = 13) in addition to examining samples individually. Across the region, average coverage was 170× in the mutant pool and 280× in the carrier pool. In the mutant and carrier pools 44 and 29 kb respectively had fewer than eight reads, a significant improvement from single‐sample coverage. Linkage was determined using allele frequency cutoffs (alternate allele divided by total reads) of at least 0.8 to call a variant as different from reference sequence with homozygous alternate alleles (for mutants), between 0.2 and 0.8 for a heterozygous call (carriers) and less than or equal to 0.2 for a homozygous call matching reference sequence (wt), as recommended in Nielsen *et al.* (2011). Selection of variant subsets and annotation with data from dbSNP (NCBI, build 151) was accomplished using snpsift (Cingolani *et al* *.* 2012b). The physiological outcomes of these variants were predicted using  snpeff (Cingolani *et al.* 2012a). The presence of structural variants was also queried using delly (Rausch *et al.* 2012) and the output was further manipulated for viewing using bcftools (Li *et al*. 2009).

## Results

### Mapping

Selection of the SNPs from the 600K array results that showed a common homozygous genotype in all nine mutants from the UCD cpp.003 line (2011 generation) as compared with the two UCD 003 normal (wt) birds, thereby suggesting linkage to *cpp*, indicated an associated region of 5.1 Mb on GGA 11. This region was delineated by 1172 informative SNPs, several of which were then used for fine‐mapping. No other *cpp*‐linked regions were found, although six SNPs were detected elsewhere in the genome: one on GGA 1, one on GGA 12 and four which could not be mapped to a specific chromosome. Such high‐resolution mapping results highlight a key benefit of using a congenic inbred line in genomic studies, as only a small number of individuals were needed to distinguish the mutation‐linked region from the inbred background genome. Several generations of birds later, fine‐mapping began with an initial examination in a small test set of 2014 generation birds at six SNPs throughout the linked region (Table S1). The results indicated a loss of linkage at the 5′ end in one of the two carrier birds examined (at SNPs rs315826859 and rs14018208). Subsequent genotyping of the entirety of the 2015 carrier generation was performed with the outermost linked SNPs (rs312679914, rs312705156) at either end of the region, but no additional recombination/size reduction was observed in this cohort. The 2016 generation of carriers was examined at these same SNPs, and one bird indicated a putative recombination event at the 3′ end of the region (rs312705156, rs315889666 and rs313102275 were shown to be unlinked). As a result, the linked region was determined to encompass 4.9 Mb, a total reduction in size of 237 kb in the five generations since the birds were sampled for the 600K SNP array. This region was targeted for the capture array.

### Sequence variation

**Figure 1:**
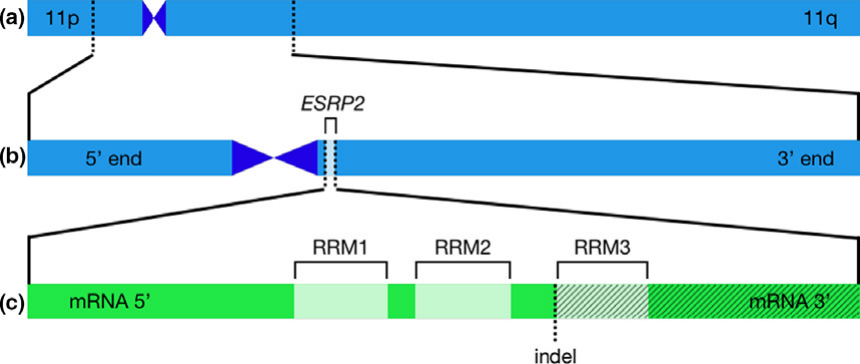
The 4.9 Mb linked candidate region of GGA 11. (a) GGA 11 with the *cpp*‐linked region (bases 1 614 496–6 488 857 in build Gallus_gallus‐5.0.86) with borders shown as dotted lines. The p (left) and q (right) arms of the chromosome are as indicated and the predicted centromere location appears as the darker constriction. (b) Expands the 4.9 Mb linked region (a) with the 5′ end (left) and 3′ end (right) orientations and the *ESRP2* gene position indicated. The other verified protein‐coding genes in the RefSeq track of the UCSC Genome Browser are listed with brief functional descriptions in Table S2. (c) Indicates the mRNA transcript of ESRP2 with 5′ and 3′ ends and the RNA recognition motifs (RRMs) indicated. The frameshift indel (ss5200091912) is marked with a dotted line at the beginning of RRM3, and the downstream transcript predicted to be mistranslated is shown by hatching.

In total, the capture array sequencing identified 23 336 SNVs and 2896 indels in the 4.9 Mb region (Table 1). Selecting for linkage to the *cpp* genotype resulted in a reduced subset of 2827 SNVs and 136 indels, located between 1 614 496 and 6 488 857 on GGA 11 (still approximately 4.9 Mb; Fig. 1a,b). This region encodes 31 protein‐coding RefSeq chicken genes per galGal5 via the UCSC Genome Browser (Table S2; O'Leary *et al.* 2016). After annotation of these linked variants with dbSNP (Sherry *et al.* 2001) build 151 data, a set of only 145 SNVs and 55 indels were observed to be unique to this *cpp* dataset, and therefore considered most likely to be potentially causative of the mutation. These variants still encompassed an approximately 4.78 Mb region (chr11:1 636 254–6 415 396) encoding the same 31 genes as in the 4.9 Mb region described above. It should be noted that Ensembl (release 94; Zerbino *et al.* 2018) has over 80 genes annotated in this same region including non‐protein‐coding genes such as those for ncRNA and miRNA, and variant overlaps with all of these are included in predicting physiological effects.

**Table 1:** Total number of variants in the 4.9 Mb candidate region of GGA 11 identified in *cpp* data by capture array sequencing.

Variant	Called	Linked to *cpp* ^1^	Linked and unique^2^ to *cpp*
SNV	23 336	2827	145
Indel	2896	136	55

^1^ Linked here means that the variant is homozygous alternate (i.e. not a match for the reference genome) in mutant samples, heterozygous in carrier samples and homozygous matching the reference genome in wt samples.

^2^ Unique variants are those not observed in dbSNP, build 151.

Structural variants (SVs), generally defined as chromosomal rearrangements such as translocations, inversions or larger insertions and deletions (greater than 50 bp; Ye *et al.* 2016), were also identified within the region. In the captured region of 4.9 Mb across all individuals, five SV deletions were predicted. However, all were flagged by the predictor tool delly (Rausch *et al.* 2012) as imprecise and the confidence as low quality (having fewer than three reads to support, or mapping quality below 20). Further, none of these SVs had the correct haplotype to be linked to the *cpp* mutation and were therefore considered unlikely for causation.

### Effect prediction

The predicted outcomes of the *cpp*‐linked variants were organized into bins. Each variant is capable of multiple consequences depending on where it overlaps with different aspects of a gene or other genomic element (i.e. the variant effect is counted rather than the variant itself), and the severity of the predicted physiological consequences of the SNVs and indels are determined after division into these categories (Table 2). The variants were primarily found in intergenic DNA, introns or up‐ or downstream of a gene (defined as 5 kb 5′ of a gene or 3′ of a gene respectively). However, one SNV effect appeared in a 3′ UTR, one SNV effect in a 5′ UTR, and three SNV and four indel effects were in exons of protein‐coding genes. The SNVs in exons created synonymous mutations, but a single indel had a strong predicted impact: a 1 bp deletion created a frameshift mutation in the gene *ESRP2*, wherein four potential transcripts were affected, accounting for the four predicted exonic effects.

**Table 2:** Variant effect locations of sequence variants unique and linked to *cpp*. The given numbers reflect all overlaps for all variants predicted by snpeff (i.e. alterations to multiple transcripts of a gene count as multiple effects). The locations are based on their annotation in the Gallus_gallus‐5.0.86 database provided with snpeff (Cingolani *et al* *.* 2012a).

Variant	Intergenic^1^	Intronic	Up‐ or downstream of a gene^2^	5′ UTR	3′ UTR	Exonic^3^
SNV	94	64	80	1	1	3
Indel	36	31	10	0	0	4

^1^ Intergenic here defined as >5 kb away from a gene.

^2^ Up‐ or downstream of a gene here means <5 kb 5′ or 3′ of a gene respectively.

^3^ All exonic variant effects were silent (i.e. synonymous) except for the four caused by one single‐base deletion (indel) that is predicted to cause frameshift mutations in four transcripts of the gene *ESRP2* (further detail in Results, Discussion).

## Discussion

Detailed characterization of the *cpp* craniofacial phenotype has been available for many years (Yee 1976; Yee & Abbott 1978; MacDonald *et al.* 2004). Here we sought to enhance the genetic and genomic knowledge of the *cpp* mutation for eventual determination of the causative element which will contribute to vertebrate biology and pathway analyses of craniofacial development. We mapped the location of the *cpp* mutation to a region of GGA 11, identified SNPs for genotyping, created a high‐confidence set of potentially causative variants linked and unique to *cpp* and identified a strong candidate variant for future studies.

GGA 11 is 20.2 Mb in size (UCSC Genome Browser; Kent *et al.* 2002), acrocentric and the largest of the chicken microchromosomes (Fig. 1a) (Axelsson *et al.* 2005). The *cpp* region of interest (galGal5 chr11:1 614 496–6 488 857; Fig. 1a,b) is near the terminus of the p arm, spans the predicted placement of the centromere (synthetically set at 0.5 Mb in size, see NCBI Assembly ID 59581; galGal5 chr11:2 804 945–3 304 944 in Fig. 1a) and continues into the q‐arm. The GGA 11 centromere has been described for its tandem repeat sequences and motifs (Shang *et al* *.* 2010), and its predicted placement in galGal5 is corroborated by ChIP‐seq data identifying peaks of enrichment for the centromeric repeat in the same region (Piegu *et al.* 2018). In any case, presuming that either the centromere is correctly placed or, if not, is at least nearby, the phenomenon of centromere interference in recombination might prevent disruption of the *cpp* region despite the high recombination rate predicted for chicken microchromosomes (6.4 cM/Mb; Burt 2005). This is supported by the minimal change in the region size observed over five generations; given this, an emphasis on variants and genes of interest will probably be a more productive route for further advancements rather than fine‐mapping.

Our analysis ultimately highlighted only one *cpp*‐linked unique variant with a strong predicted physiological effect. This single‐base deletion (galGal5 chr11:3 384 959; ss5200091912) is predicted to cause a frameshift mutation in *ESRP2* affecting multiple transcripts (Fig. 1c). The deletion is identified in exon 11 or 12 and amino acid 443 or 451 depending on the transcript, which places it near the beginning of the third RNA recognition motif in the gene (https://www.uniprot.org/uniprot/Q5ZLR4; The UniProt Consortium 2018). Altered DNA in this region disrupting the sequence‐specific motif is therefore likely to affect ESRP2 protein function. *ESRP2* is a splicing regulator, and along with *ESRP1*, specifies the IIIb isoform of *FGFR2* in epithelial cells vs. the IIIc isoform found in mesenchymal cells in humans (Warzecha *et al.* 2009a; GeneCard ID: GC16M068229; Stelzer *et al.* 2016). Notably,  *fibroblast growth factor receptor* (*FGFR*) genes are involved in cell proliferation and differentiation regulation during development (Tiong *et al.* 2013). Further, the *cpp* defect is specifically found in the epithelial tissue of the frontonasal prominence, which exhibits abnormal *Fgf8* mRNA expression (MacDonald *et al.* 2004). FGFs are also involved in regulating cilia length and function (Neugebauer *et al.* 2009; Brugmann *et al.* 2010), and it is of note that two craniofacial mutations in chicken have already been classified as models for ciliopathies (*talpid2* and *talpid3*; Schock *et al.* 2016). Interestingly, scanning electron microscopy images indicated that *cpp* mutants lack the long filaments that span the distance between maxillary and mandibular processes (MXP and MNP) immediately prior to MXP–MNP fusion in normal embryos (Yee & Abbott 1978).

The *ESRP* genes in mammals are key regulators of alternative splicing during the epithelial to mesenchymal transition and have an expansive set of targets beyond *FGFR2* (Warzecha *et al.* 2009b). Mouse KOs of Esrp1 develop cleft lip and palate and are neonatal lethal 1 day after birth, and ablation of both Esrp1 and Esrp2 resulted in more severe craniofacial defects as well as abnormal limbs, agenesis of lungs and salivary glands, thinner skin with reduced follicles and reduced kidneys (Bebee *et al*. 2015). The chicken *cpp* mutant phenotype is syndromic, with some similarity to the mouse double KO in that *cpp* mutants fail to develop a normal respiratory system (lungs and air sacs), as well as metanephric (adult) kidneys (Yee 1976). Although we propose that an altered ESRP2 protein may be responsible for the *cpp* phenotype, an Esrp2 KO alone in mice does not have a phenotypic effect (Bebee *et al*. 2015). While this suggests that the relative roles of *ESRP1* and *2* may not be identical in chicken and mouse, the overall disruption of these genes has strong phenotypic similarities across multiple developmental systems. The mammalian *ESRP1/2* have significant sequence conservation, particularly in RNA recognition motifs with chicken and other species (Warzecha *et al.* 2009a), but divergence between their downstream targets may be a source of distinction between these genes’ activity in mammals and other vertebrates.

We have made significant strides in improving the genome biology of the *cpp* mutation, a valuable model system for studying vertebrate facial development. We mapped the mutation to GGA 11 within a 4.9 Mb chromosomal region extending from the p arm, through the centromere and into the q arm, identified linked SNPs that can be used to genotype individuals from the UCD cpp.003 line and assessed the variation in the linked region. The prioritized list of potentially causative variants will be further refined as new variant data and better genomic annotations become available for comparison to our dataset. Future work will assess the expression of the genes in the linked region, and herein we propose a specific focus on *ESRP2* as the top priority candidate owing to the predicted frameshift mutation identified by our work.

## Supporting information


**Table S1** PCR primers used for SNP genotyping of the *cpp* mutation candidate region in UCD cpp.003 individuals.
**Table S2** Protein‐coding chicken genes in the 4.9 Mb linked *cpp* mutation candidate region on GGA 11.

**Table S3** *Cpp* SNV accessions.

**Table S4** *Cpp* indel accesions.

## Data Availability

Read data from the capture array are available via BioProject ID PRJNA547998 at NCBI SRA. Variants found to be linked and unique to cpp are available via Project Accession PRJEB33252 at EMBL‐EBI. Sequence accession numbers for cpp‐SNVs and cpp‐Indels are found in Table S3 and S4, respectively.
