# Supplementary material for: Mapping of the chicken cleft primary palate mutation on chromosome 11 and sequencing of the 4.9 Mb linked region
Source: Anim Genet. 2020 Mar 12;51(3):423–9. doi: 10.1111/age.12927 (PMC7317479; doi:10.1111/age.12927)
Supplement: Supplementary file 1 — Table S1 PCR primers used for SNP genotyping of the cpp mutation candidate region in UCD cpp.003 individuals. Table S2 Protein‐coding chicken genes in the 4.9 Mb linked cpp mutation candidate region on GGA 11. [file AGE-51-423-s001.docx]

Supplemental Table 1. **PCR primers used for SNP genotyping of the *cpp* mutation candidate region in UCD cpp.003 individuals.**

| **rsID of SNP^1^** | **Forward primer (5' to 3')** | **Reverse primer (5' to 3')** | **SNP location^2^** | **Product size (bp)** | |
| --- | --- | --- | --- | --- | --- |
| 315826859 | CCTTTTTCGTATCAGTTCT | TAATACTGTGGGTAACAAAG | 1479640 | | 324 |
| 14018208 | CAATGTATTTACCAGTTGTG | ATATGACCTCATCCTTCATA | 1612981 | | 301 |
| 312679914 | CTCATTTATGTTCTGGTTAC | ATTTCTGGCTTATTTTTCCT | 2295221 | | 344 |
| 314265637 | ACAATTAAAGATCTGGAACA | CATATGCAAAGGAATTTAGC | 3649468 | | 319 |
| 318225998 | AGAGAACAGAAGGTTTATTT | GGTATATGGCTTTTCTTCTT | 5124583 | | 359 |
| 315889666 | ATAAATGTACCTATTCTTAC | AGGATTTTCACTGTAATG | 6555178 | | 379 |
| 315690912 | TTTTGGTTAATAAGCGAACAC | GGTTGCCCTCTGGAT | 2071367 | | 400 |
| 316449172 | AAGGAACTCCATCATCAAT | ATAATGCTTTTCCCTTCATA | 2396244 | | 300 |
| 313102275 | CTACGTTAGCAAAGAACTAT | TTTATTCGTCAACTATGC | 6503406 | | 370 |
| 312705156 | CATTAACTGCAATTCAGAG | CTTACTGTTTCTGTTGGAA | 6606629 | | 400 |

^1^SNPs are taken from the 600K array used for initial mapping of the *cpp* mutation. Each numbered ID here is preceded by “rs”.

^2^Coordinates are in GGA 11, genome build Gallus_gallus-5.0.Supplemental Table 2. **Protein-coding chicken genes in the 4.9 Mb *cpp* mutation candidate region on GGA 11.**

| Gene^1^ | NCBI ID | Name | UniProt ID | Function^2^ |
| --- | --- | --- | --- | --- |
| HYDIN | 427538 | hydrocephalus-inducing protein homolog | F1NYR0 | required for ciliary motility |
| VAC14 | 415678 | protein VAC14 homolog, PIKFYVE complex component | Q5ZIW5 | involved in synthesis and turnover of phosphatidylinositol 3,5-bisphosphate, a positive activator of PIKfyve kinase activity |
| COG4 | 415681 | component of oligomeric Golgi complex 4 | F1NHU0 | associated with Golgi organization and vesicle prefusion complex stabilization, and Golgi to ER retrograde vesicle-mediated transport |
| ST3GAL2 | 395139 | ST3 beta-galactoside alpha-2,3-sialyltransferase 2 | F1NGY0 | associated with glycolipid and oligosaccharide biosynthetic process, protein glycosylation and sialylation |
| GLG1 | 396492 | Golgi glycoprotein 1 | Q02391 | binds cell-adhesion lectin on endothelial cells, binds FGF, possible involvement in intracellular FGF trafficking and regulating cellular responses to FGFs |
| WDR59 | 415688 | WD repeat domain 59, GATOR complex protein | Q5ZLG9 | part of the GATOR complex, may be part of the amino acid-sensing branch of the TORC1 signaling pathway |
| CTRB2 | 431235 | chymotrypsinogen B2 | H9KYN7 | associated with serine-type endopeptidase activity |
| CFDP1 | 374073 | craniofacial development protein 1 | Q75QI0 | may play a role during embryogenesis, associated with multicellular organism development |
| TMEM170A | 770531 | transmembrane protein 170A | Q5ZM31 | may regulate membrane morphogenesis in the ER via promoting ER sheet formation using ER tubules |
| NUP93 | 415693 | nucleoporin 93 | A0A1D5PFL9 | plays a role in the nuclear pore complex assembly and/or maintenance |
| MT4L | 396212 | metallothionein 4-like | PT47944 (human) | (in human:) appears to bind zinc and copper, may play a role in regulating zinc metabolism during stratified epithelia differentiation |
| MT3 | 770592 | metallothionein 3 | Q98947 | associated with zinc ion binding and metalloendopeptidase activity |
| BBS2 | 415694 | Bardet-Biedl syndrome 2 | Q5ZI17 | associated with RNA polymerase II repressing transcription factor binding and non-motile cilium assembly |
| NUDT21 | 100858636 | nudix hydrolase 21 | E1C538 | associated with chromatin, histone deacetylase, AU-rich element, and mRNA binding; also hydrolase activity and protein homodimerization activity |
| GNAO1 | 415698 | G protein subunit alpha o1 | E1C347 | associated with binding of corticotropin-releasing hormone receptor 1, G-protein-coupled receptor and serotonin receptor, mu-type opioid receptor, and GTP; also GTPase activity |
| CBFB | 395297 | core-binding factor beta subunit | Q8QGE8 | associated with transcription coactivator activity |
| C11H16orf70 | 415699 | chromosome 11 open reading frame, human C16orf70 | Q5ZKM4 | associated with Golgi to plasma membrane protein transport |
| FBXL8 | 415701 | F-box and leucine rich repeat protein 8 | F1NNN0 | associated with protein ubiquitination and the SCF-dependent proteasomal ubiquitin-dependent protein catabolic process |
| HSF4 | 427540 | heat shock transcription factor 4 | F1NNK6 | associated with DNA-binding transcription factor activity, sequence-specific DNA binding, and RNA polymerase II proximal promoter sequence-specific DNA binding |
| HSDL1 | 415703 | hydroxysteroid dehydrogenase like 1 | Q5ZJG8 | may catalyze steroid hormone metabolism important to sex differentiation, secondary sexual characteristics (emergence/maintenance), and endocrine regulation |
| ESRP2 | 415710 | epithelial splicing regulatory protein 2 | Q5ZLR4 | mRNA splicing factor that regulates the formation of epithelial cell-specific isoforms (i.e., FGFR2-IIIb), specifically binds sequences in mRNAs, GU-rich sequence motifs in ISE/ISS-3 (cis-element regulatory region in FGFR2 mRNA) (based on similarity to human) |
| GALR1L | 415713 | galanin receptor type 1-like | F1NQJ8 | associated with galanin and G protein-coupled peptide receptor activity |
| SLC6A2 | 395458 | solute carrier family 6 member 2 | Q9DGN5 | associated with neurotransmitter sodium symporter activity and positive regulation of peptide hormone secretion |
| LPCAT2 | 415716 | Lysophosphatidylcho-line acyltransferase 2 | F1NJT9 | associated with 1-acylglycerophosphocholine O-acyltransferase activity and calcium ion binding |
| MMP2 | 386583 | matrix metallopeptidase 2 | Q90611 | has catalytic activity in cleavage of gelatin type I and collagen types IV, V, VII, and X |
| FTO | 415718 | FTO, alpha-ketoglutarate dependent dioxygenase | A0A1D5PX99 | associated with activity of DNA-N1-methyladine dioxygenase, oxidative DNA demethylase, tRNA demethylase, and RNA N6-methyladenosine dioxygenase; also ferrous iron binding |
| AKTIP | 415720 | AKT interacting protein | Q5ZJJ5 | may function in promotion of vesicle trafficking and/or fusion, may regulate apoptosis (based on similarity to human) |
| RBL2 | 425111 | RB transcriptional corepressor like 2 | F1NEZ1 | associated with regulation of cell cycle, division, and transcription by RNA polymerase II |
| TOX3 | 415724 | TOX high mobility group box family member 3 | A0A1D5PEI4 | associated with DNA binding |
| SALL1 | 395446 | spalt like transcription factor 1 | A0A1D5PFD0 | associated with nucleic acid binding |
| SNX20 | 415726 | sorting nexin 20 | F1NIU2 | associated with phosphatidylinositol-3-phosphate and phosphatidylinsoitol-4,5-bisphosphate binding as well as protein transport |
|  |  |  |  |  |
| ^1^All genes are verified RefSeq genes in chicken, and appear on the Gallus_gallus-5.0 build on the UCSC Genome Browser. | | | | |
| ^2^Functions are taken from the short description in UniProt. | | | | |
|  |  |  |  |  |
|  |  |  |  |  |
